# Magnetic Domain Characterization and Physical Properties of Gd-Doped and (Gd, Al) Co-Doped ZnO Thin Films

**DOI:** 10.3390/ma15228025

**Published:** 2022-11-14

**Authors:** Nur Amaliyana Raship, Siti Nooraya Mohd Tawil, Nafarizal Nayan, Khadijah Ismail, Anis Suhaili Bakri, Zulkifli Azman, Faezahana Mohkhter

**Affiliations:** 1 Department of Electrical and Electronic Engineering, Universiti Pertahanan Nasional Malaysia (UPNM), Kem Sungai Besi, Kuala Lumpur 57000, Malaysia; 2 Center for Tropicalisation, Universiti Pertahanan Nasional Malaysia (UPNM), Kem Sungai Besi, Kuala Lumpur 57000, Malaysia; 3 Microelectronic and Nanotechnology-Shamsuddin Research Centre (MiNT-SRC), Universiti Tun Hussein Onn Malaysia (UTHM), Parit Raja 86400, Malaysia; 4 Faculty of Electrical and Electronic Engineering, Universiti Tun Hussein Onn Malaysia (UTHM), Parit Raja 86400, Malaysia

**Keywords:** Gd-doped ZnO, shallow donor, magnetic force microscopy, co-doping, sputtering

## Abstract

Undoped ZnO, Gd-doped ZnO with various doping concentration (1, 3, 5, and 7 at%), and 3 at% (Gd, Al) co-doped ZnO films were prepared on a glass substrate using the co-reactive sputtering method. The influence of the doping and co-doping process on the films was characterized using X-ray diffraction, FESEM, EDX, MFM, VSM, UV–VIS spectroscopy, and the Hall Effect measurement at room temperature. XRD study confirmed that the Gd and Al ions are incorporated into a ZnO lattice. EDX analysis confirmed the existence of Zn, O, Al, and Gd elements in the prepared Gd-doped ZnO and (Gd, Al) co-doped ZnO films, which suggests the successful doping procedure. All the deposited films obtained maximum optical transmittance above 80%, showing a high transparency of the films in the visible region. The optical band gap was found red-shifted from 3.11 to 3.21 eV with the increase in Gd doping concentration. The increase in band gap energy from 3.14 eV to 3.16 eV was obtained for 3 at% Gd and 3 at% (Gd, Al) co-doped ZnO films. The MFM measurement proved the existence of room-temperature ferromagnetism and spin polarization in Gd and (Gd, Al) co-doped ZnO films. By co-doping with Al, the result obtained from MFM shows the enhancement of magnetic properties, as it exhibited a smaller domain size with a shorter magnetic correlation length *L*, a larger phase shift Φ_rms_, and the highest value of *δf*_rms_ compared to the sample with 3 at% Gd incorporated into ZnO. The carrier concentration and electrical conductivity increased with the increase in Gd concentration, whereas the electrical resistivity and hall mobility showed a reverse trend. The similar trend of results obtained for 3 at% (Gd, Al) co-doped ZnO as compared to 3 at% Gd-doped ZnO also indicates greater electrical properties after a shallow donor such as aluminum was incorporated into Gd-doped ZnO thin films. In conclusion, for future applications, one should consider the possible influence of other types of shallow donor incorporation in an attempt to enhance the properties of new types of diluted magnetic semiconductors (DMSs).

## 1. Introduction

The utilization of nano-devices by semiconductor materials is becoming increasingly popular and demanding. New inventions of various applications with new functionalities can be achieved through diluted magnetic semiconductors (DMSs) by combining the use of both the charge and spin of electrons. Among the semiconductor-based DMSs, the zinc oxide (ZnO)-based DMS has attracted great attention, since the resulting materials can display a good ferromagnetism with a Curie temperature above room temperature. ZnO is categorized in n-type semiconductors by having a direct wide band gap of 3.37 eV, a high exciton binding energy of 60 meV, and high electron mobility. The doping transition or rare-earth metals into ZnO, in general, is an effective technique to improve optical, electrical, and magnetic properties. Enhancing the properties of ZnO through doping makes it suitable for various applications such as sensors, microwave devices, solar cells, high-density magnetic-optical storage media, and spintronics [1]. 

In recent years, most of the interest in rare-earth ions such as Gd, Eu, Tb, and Nd is due to their high magnetic moment and because these materials exhibit a room-temperature ferromagnetism [2,3,4,5,6]. Amongst rare-earth ions, Gd produces stronger ferromagnetism since it has an unpaired 4f shell of electrons and a polarized 6s and 5d valence [7]. Gd, as a dopant element, is also a promising material especially for the growth of durable magnets because of its great intrinsic magnetic properties. Moreover, materials that display extended ferromagnetism are appropriate for the new advanced technology of spin-based electronic devices. Co-doping is considered to be the extension of ferromagnetism by specifying available carriers. It is thought that the existence of supplementary carriers brought in by co-doping can further enhance ferromagnetism in ZnO. The improvement of carrier-mediated ferromagnetism and high saturation magnetization at room temperature by co-doping with shallow donors and acceptors has been observed in other types of DMS systems [8]. Saleem et al. [9] observed the enhancement of ferromagnetism in (Ni, Al) co-doped ZnO, where the co-doping of Al enhances the free carriers in ZnO. Likewise, in (ZnO, Co) co-doped with Gd, reported by Thangeeswari et al. [10], the measurement from magnetic properties shows that co-doping produces a room-temperature ferromagnetism, while Gd co-doping has the ability to enhance the magnetic moment. More recently, Ghoul et al. [11] reported enhanced room-temperature ferromagnetism behavior due to (V, Gd) co-doping in ZnO. The above outcomes suggest that co-doping is a promising way to enhance the ferromagnetic properties of doped ZnO systems.

To date, how the doping element’s concentration influences the magnetic microstructure and how physical properties correlate with the magnetic microstructure and electrical properties are still uncertain. In this study, Gd-doped ZnO and (Gd, Al) co-doped ZnO films were prepared using the co-sputtering technique, which is a technology that can provide optimum coating uniformity, can easily control the stoichiometry of the sample, has a high deposition rate, and is suitable for large-scale deposition. The influence of Gd and Al concentration on the film’s physical properties and electrical properties was investigated, and how Gd and Al doping affects the magnetic domain structures of ZnO films probed via Magnetic Force Microscopy (MFM) was also discussed.

## 2. Materials and Methods

**Figure 1:**
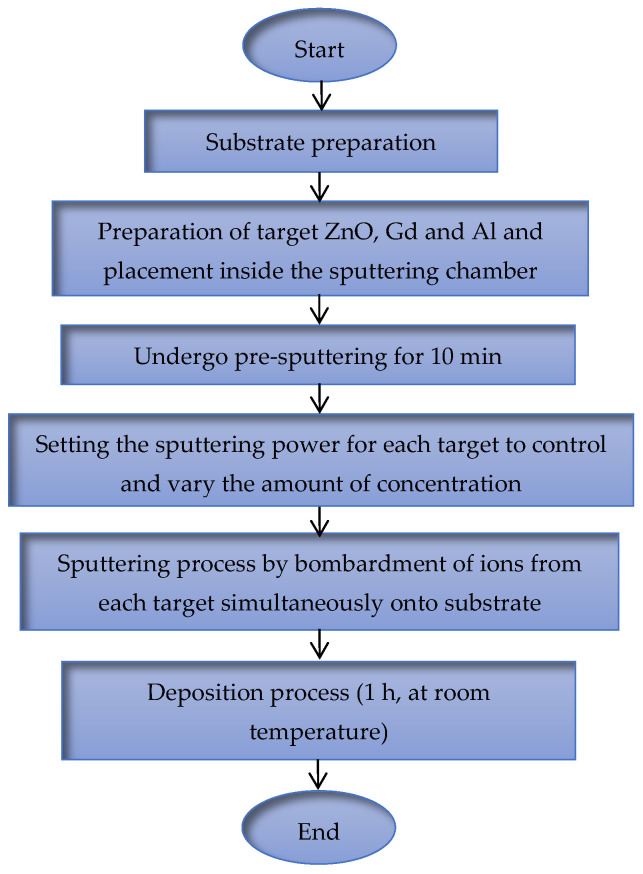
Flow chart of deposition process of undoped ZnO, Gd-doped ZnO at various Gd concentrations, and (Gd, Al) co-doped ZnO.

The undoped ZnO, Gd-doped ZnO, and (Gd, Al) co-doped ZnO films were deposited on glass substrate using a magnetron sputtering system (SINTEK PSP 5004 (09SN70)) by a co-sputtering process [12,13]. The cleaning process for the glass substrates was carried out before the deposition process, as reported in previous study [14]. For deposition purposes, the ZnO target (99.99%), Gd target (99.99%), and Al target (99.99%) were connected to DC, RF, and RF power supply, respectively. First, a turbo molecular pump (TMP) was used to evacuate the base chamber pressure to 5.0 × 10^−6^ Torr. Next, argon gas with the flow rate set constant at 29 sccm was introduced for the deposition process. Pre-sputtered target was initiated for 15 min in order to remove the impurities on the target surface. The sputtering power and working pressure of the ZnO target were retained at 100 W and 10 mTorr, respectively. The distance from the target to the substrate was fixed at 13.5 cm. In order to endorse uniformity of the deposited films, the glass substrate was set to rotate consistently at 9 rpm throughout the deposition process. The deposition process was conducted at room temperature for 60 min. In this work, the concentrations of Gd were varied at 0, 1, 3, 5, 7 at%, whereas 3 at% was set for (Gd, Al) co-doped ZnO films. The different concentrations of samples were achieved by varying the power of the Gd target. The thickness of films obtained is about 300 nm. The flow of the deposition process of the prepared films is shown in Figure 1.

The crystal structure and phase indications of the films were measured by X-ray diffraction (XRD), using PANalytical X-Pert Powder. All the samples were scanned by setting the Cu Kα radiation wavelength as λ = 0.1540 nm with a 2θ degree angle within the range of 20–80°. The atomic percentage (at%) of the Gd and Al doping composition were assessed via EDX analysis by attaching the probe to the field electric scanning electron microscope (FESEM) using a JEOL JSM-7600F. The transmittance spectra and energy band gap of the films were determined from UV–Vis spectroscopy. The magnetic domain structures of samples were examined in detail at the nanometer scale by Magnetic Force Microscopy (MFM), with all samples scanned in dimensions of 1 μm × 1 μm. The MFM utilizes a magnetic tip to evaluate the magnetic force gradient distribution from a sample’s surface by oscillating the cantilever normal to the surface at its resonant frequency. The magnetization of the MFM tip with high lateral resolution and high coercivity is placed perpendicular to the sample surface and points downward. All the MFM images were produced at the same lift scan height of 50 nm, utilizing the tapping mode measured at room temperature. Magnetization measurements were measured using a vibrating sample magnetometer (VSM) (LakeShore 7404, Carson, CA, USA) with a maximum magnetic field of 10 kOe carried out a room temperature. Hall effect measurement (Lakeshore Cryotronics 8400 series) was performed in order to investigate the electrical properties of the films.

## 3. Results and Discussion

### 3.1. XRD Analysis

**Figure 2:**
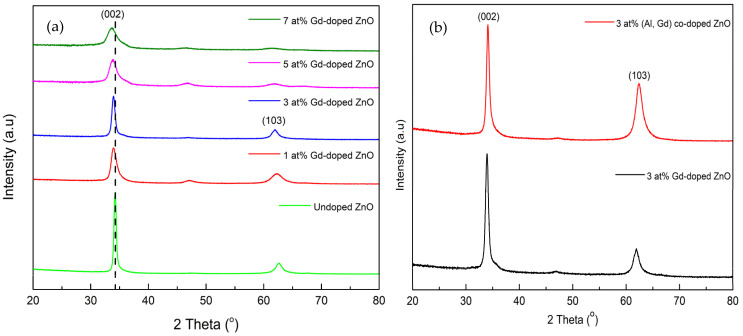
XRD spectra of (**a**) undoped ZnO and Gd-doped ZnO films at various Gd concentrations, (**b**) 3 at% Gd-doped ZnO films and 3 at% (Gd, Al) co-doped ZnO films.

In order to obtain a better understanding of the structural properties and identify the phases of the films, X-ray diffraction analysis was carried out. Figure 2a shows the X-ray diffraction pattern for the undoped ZnO and (1, 3, 5, 7 at%) Gd-doped ZnO films. The diffraction crystal planes of (002), (102), and (103) were observed, in which the peaks are located at 2θ of ~34.0°, ~47.0°, and ~62.0°, respectively. These two crystal planes refer to a pure ZnO phase with a hexagonal wurtzite structure coordinated with the database from Highscore software for an ICSD number of 98-018-6243. The dominant peak was observed at the (002) plane, showing that the films have a c-axis preferred orientation. The same observations were obtained from the 3 at% (Gd, Al) co-doped ZnO film, as shown in Figure 2b. Moreover, no secondary phases or additional peaks were observed in the ZnO doped with Gd or co-doped with Al. This further indicates that Gd^3+^ and Al^3+^ ions were substituted into Zn^2+^ ions, which consequently justifies the incorporation of dopants into the ZnO lattice of interstitial sites without distorting the crystal structure of the ZnO itself [15,16]. This is also ascribed to the smaller ionic radius of Al^3+^ (0.54 Å) compared to the Zn^2+^ (0.74 Å), hence the aluminum ions are easily incorporated into the host zinc sites. In comparison with undoped ZnO, the XRD peak of (002) with an increased Gd concentration has slightly shifted to a lower diffraction angle, as indicated by the dash line in Figure 2a. This was mostly due to the different ionic radius of the Gd ions incorporated into the ZnO lattice, the Gd ions having a greater ionic radius of 0.93 Å than the host ZnO of 0.74 Å [17]. As reported by Yusoff et al., the incorporation of ions with a large ionic radius shifts the XRD diffraction to a lower angle, whereas ions with a smaller ionic radius move the XRD diffraction peak to a higher angle [18]. Increasing Gd doping up to 7 at% also does not change the standard ZnO wurtzite structure, which consists of alternating planes of tetrahedral-coordinated O^2−^ and Zn^2+^ ions stacked along the c-axis. Concurrently, the increase in Gd doping concentration reduced the intensity of the (002) peak, which indicates the change of crystallite orientation at a higher Gd content. The XRD peak of (002) at higher Gd concentrations also seems to shift slightly towards the lower diffraction angle in contrast to that of the undoped ZnO. The shifted diffraction peak is mainly due to the substitution of Gd ions (0.93 Å), which have a larger ionic radius than the host ZnO (0.74 Å), inside the structure of the hexagonal wurtzite [17,19]. This signifies that the Gd atoms substitute Zn atoms, and the crystal structure remains unchanged. The analysis of (002) XRD peaks for Gd-doped ZnO at various Gd concentrations is tabulated in Table 1. The crystallite size (D) of all the films was calculated using Debye–Scherrer’s equation based on the FWHM value computed from XRD software analysis, which is given by:

D_(002)_ = (0.9 λ)/(β cos θ) (1)

where D is the crystallite size of the particle, λ is 0.154 nm of the incident X-ray wavelength, and β is the full width at half maximum (FWHM) of the observed peak, which is the (002) peak for this study. A clear pattern was observed from the decrease in the crystallite size with the increasing amount of Gd, as indicated by the sample with Gd 3 at%, Gd 5 at%, and Gd 7 at%. 

With the increase in Gd concentration, the crystallite size decreased. This is believed to be due to the incorporation of Gd ions into the ZnO lattice, which contributes to distortions of the lattice. Generally, the lattice distortion occurred because Gd has a higher ionic radius, which interrupts the lower ionic radius of Zn [20]. Note that the increase in crystallite size indicates the improvement in crystallinity of the films [21]. This finding exhibits that the solubility limit of Gd ions into the ZnO crystal lattice is at 3 at% and an excess of Gd ions may deteriorates the crystal quality of the films. Similar observations have been reported by Yi et al. [22].

Furthermore, the full width at half maximum (FWHM) value of the (002) peak shows an increment with the increase in Gd concentration to 7 at%. Meanwhile, the increase in Gd concentration deteriorates the (002) peak intensity, as well as the (103) peak intensity. This implies that increasing the Gd concentration could modify the crystal quality of the film [23]. On the other hand, the FWHM for 3 at% (Gd, Al) co-doped ZnO film is smaller compared to 3 at% Gd-doped ZnO. In general, the smaller FWHM value denotes an enhancement of crystal quality. This indicates that Al co-doping enhanced the crystal quality of the ZnO structure. 

The dislocation density (δ) and microstrain (ε) were calculated using Williamson and Smallman’s equation, as shown below, where the crystalline defects of the films can be determined:

δ = 1/D^2^ (2)

ε = (β cos θ)/4(3)

where D is the crystallite size, β is the (002) peak’s full width at half maximum (FWHM), and θ is the Bragg angle corresponding to the (002) peak. The dislocation density and microstrain values increase when both Gd and Al were incorporated into ZnO. This indicates the improvement of defects in the crystal structure of Gd-doped ZnO and the enhancement by Al co-doped compared to undoped ZnO, which is deduced by the lattice misfit [2,24].

### 3.2. Surface Morphology and EDX Analysis

**Figure 3:**
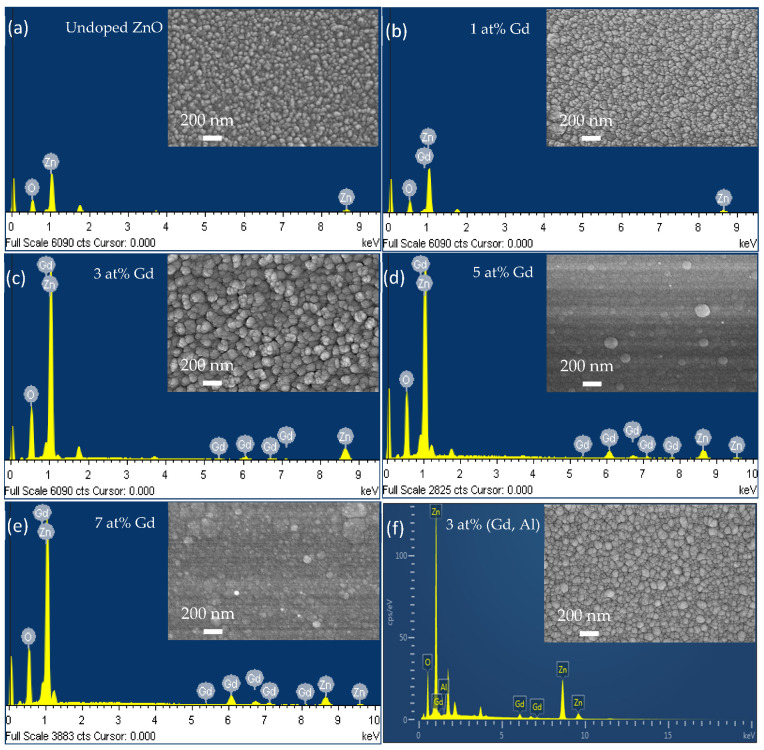
EDX spectrum of (**a**) undoped ZnO, (**b**–**e**) Gd-doped ZnO film at various Gd concentrations, and (**f**) 3 at% (Gd, Al) co-doped ZnO films. The inset figure shows the FESEM.

The composition of each Gd, Al, Zn, and O for undoped ZnO, Gd-doped ZnO, and (Gd, Al) co-doped ZnO films measured using a FESEM machine equipped with EDX spectroscopy is presented in Figure 3. The EDX spectrum reveals the existence of particular compositions of Zn, O, Gd, and Al in the film. Based on the resulting spectrum, there are no impurities or other composites traced that further confirm the successful incorporation of Al and Gd ions into the Zn host sites. This result is in agreement with the XRD result, as no other phases or secondary peaks were detected. 

In Figure 3a, there is the presence of only Zn and O, since the films are undoped ZnO, which is 0 at%, whereas in Figure 3b, there is a Gd peak detected for Gd-doped ZnO films with 3 at%. In addition, an Al peak was observed for (Gd, Al) co-doped ZnO films, which confirms the incorporation of Al ions into ZnO ions, as shown in Figure 3f. EDX was also tested at various parts of the sample and the result acquired is almost the same for the whole surface of the film, demonstrating the homogeneous and consistent distribution of Gd, Al, Zn, and O in undoped ZnO, Gd-doped ZnO, and (Gd, Al) co-doped ZnO thin films. The elemental composition analysis of Gd, Al, Zn, and O determined from EDX spectra is tabulated in Table 2.

The inset figure in Figure 3 shows the FESEM images of the prepared films. The FESEM images show that undoped ZnO and Gd-doped ZnO films have a nanoparticle structure. Based on the FESEM images in the inset of Figure 3, films that deposited up to 3 at% Gd were found to have a homogeneous and consistent surface shape. However, ZnO doped with 5 at% and 7 at% Gd concentration in the inset of Figure 3d,e, does not exhibit well-defined spherical particle shapes. The spherical structures were shattered as the Gd concentration increased. Moreover, increasing the Gd concentration reduces the size of the nanoparticles structure, as seen in the inset of Figure 3d,e. A similar pattern of results was reported by Aparna et al. [20]. This result is consistent with the XRD findings, as the crystallite size also decreased with Gd concentration. 

### 3.3. UV–Vis Analysis

**Figure 4:**
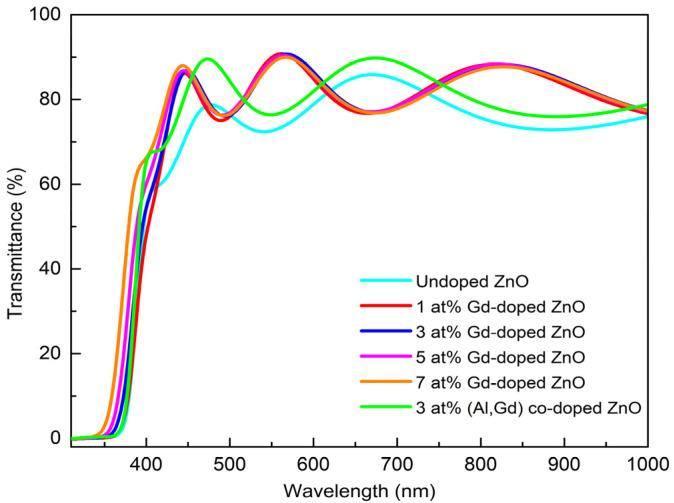
The transmittance of undoped ZnO, Gd-doped ZnO films with various Gd concentrations, and 3 at% (Al, Gd) co-doped ZnO films.

To study the optical properties of the films, the UV–Vis was used to measure the optical transmittance, as well as band gap energy, of Gd-doped ZnO thin films. The transmittance spectra of undoped ZnO, Gd-doped ZnO films with different Gd concentrations, and 3 at% (Gd, Al) co-doped ZnO are shown in Figure 4. The transmittance spectra of undoped ZnO, Gd-doped ZnO (1, 3, 5, 7 at%), and 3 at% (Gd, Al) co-doped ZnO films were tested in the wavelength range of 300 nm to 1000 nm. Based on the transmittance spectra, all films show a very high transparency in which the transmittance is above 80%, close to the infrared range in the visible area. This indicates that the films are of good transparency, can usually be used and are good enough for optoelectronics application [23]. All the transmittance spectra also have the highest and lowest spectra in the area of the visible range because of the effects from multiple interference. These effects observed are a result of light reflectance between the films themselves and the interface of the substrate, which indicates the high crystal quality of the films [14,25].

Based on Figure 4, the highest rebound of the transmittance spectra is observed for 3 at% (Gd, Al) co-doped ZnO, for which the maximum exceeds 90.8% and the average transmittance value is 82.9%. These data correlate with the results from the structural properties inferred from XRD, showing that good transmittance produces films with a good crystallinity [26]. The position of the absorption edges was observed around 350 nm to 380 nm. Moreover, the sharp absorption close to the band edges was observed for all deposited films (Figure 4), signifying no existence of other phases with the addition of Gd concentrations [25]. It was noticed that the absorption edges have a blueshift, meaning that the position shifts approaching the shorter wavelength region with the increase in Gd concentration. This phenomenon indicates an increase in the optical band gap with an increasing Gd concentration. The optical band gap energy (E_g_) of undoped ZnO, (1, 3, 5, 7 at%) Gd-doped ZnO, and 3 at% (Gd, Al) co-doped ZnO films was obtained from the transmittance spectra by using the Tauc model and parabolic bands and calculated using the following equation:

(4) $$\alpha hv=A{\left(hv-E_{g}\right)}^{n}$$

where the value *A* is consistent, α is the absorption coefficient, *hv* is the photon energy, *E_g_* is the band gap energy, and n is a constant (*n* = 0.5 is the direct band gap, whereas *n* = 2 is the indirect band gap). ZnO is generally classified as a direct band gap material; thus, the constant *n* = 0.5 is set for these calculations [27]. 

**Figure 5:**
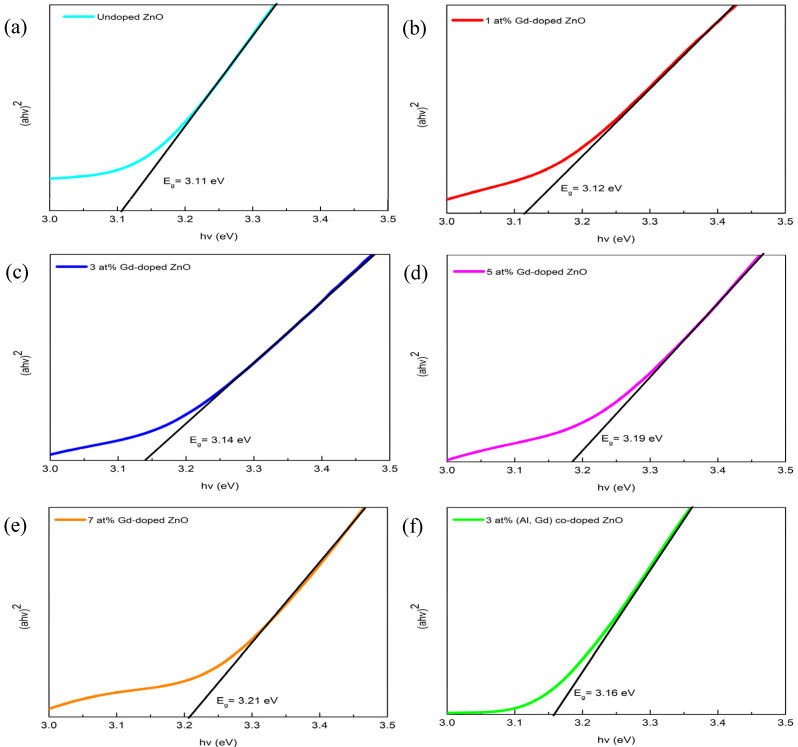
Graph of (αhν)2 versus hν for (**a**) Undoped ZnO, (**b**–**e**) Gd-doped ZnO films, and (**f**) 3 at% (Gd, Al) co-doped ZnO films.

The plotting of (αhν)^2^ versus hν for undoped ZnO, (1, 3, 5, 7 at%) Gd-doped ZnO, and 3 at% (Gd, Al) co-doped ZnO films is shown in Figure 5. The band gap values of undoped ZnO and Gd-doped ZnO films with 1, 3, 5, 7 at% Gd doping concentration were found to be 3.11, 3.12, 3.14, 3.19, and 3.21 eV, respectively. The obtained band gap values of undoped ZnO and Gd-doped ZnO films increased with the increase in Gd concentration. A similar study has been reported by other researchers [14,20]. Moreover, there is also a blueshift in the absorption edge between 3 at% Gd-doped ZnO and 3 at% (Gd, Al) co-doped ZnO, which shows the increase in band gap energy from 3.14 eV to 3.16 eV. The redshift in band gap energy was attributed to the Moss–Burstein effect [28,29]. The variation in band gap energy caused by the Moss–Burstein effect is associated to charge carrier density, which can be elucidated by the following equation:

(5) $$\Delta E_{g}=\frac{h^{2}}{8m^{*}}\;{\left(\frac{3}{\pi }\right)}^{\frac{2}{3}}\;n^{\frac{2}{3}}$$

where *h* is the Planck constant and m*(2*m*_e_) is the effective mass of electrons in the conduction band. ZnO is generally established as an n-type semiconductor because of the intrinsic donor defect, such as oxygen vacancy (V_o_) and/or zinc interstitial (Zn_i_). Free charge carriers are released within the lattice structure, when Gd and Al ions are incorporated into the ZnO wurtzite structure by replacing Zn ions. The donor electrons in Gd and Al doping generally occupy the lowest conduction band state. Based on the Pauli principle, those states are restricted to become doubly occupied. Due to the vertical nature of the optical transition, these valance electrons now require additional energy to be excited into the higher energy state in the conduction band, causing the energy band gap to widen. Therefore, the significant increase in band gap energy for Gd-doped ZnO and (Gd, Al) co-doped ZnO films is due to an ascending shift of the absorption band edge toward high photon energy.

### 3.4. MFM Studies

**Figure 6:**
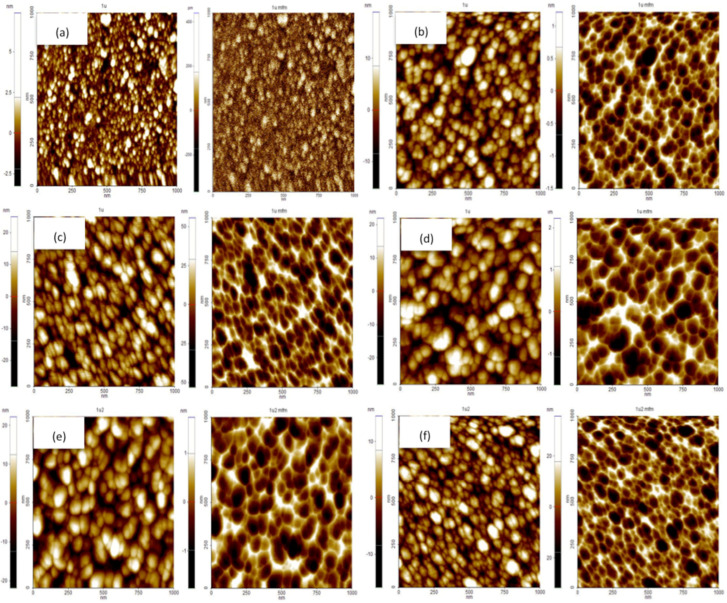
2D AFM images (left) and 2D MFM images (right) of (**a**) undoped ZnO, (**b**) 1 at%, (**c**) 3 at%, (**d**) 5 at%, (**e**) 7 at% Gd-doped ZnO, and (**f**) 3 at% (Gd, Al) co-doped ZnO films.

The magnetic properties were probed by means of magnetic force microscopy (MFM) measurements through observing the magnetic domain structure of the films. MFM is where the magnetic films are scanned using magnetic probes and the interaction among the probe itself with the films is detected, which recreates the magnetic structure of the film surface [30]. The MFM measurement was performed with a scanning dimension of 1 μm × 1 μm with a lift tip distance of 50 nm. The MFM images of undoped ZnO, (1, 3, 5, 7 at%) Gd-doped ZnO, and 3 at% (Gd, Al) co-doped ZnO films are shown on the right side of Figure 6, whereas the images of the AFM topography are on the left side. The observed magnetic domain structures show granular features for Gd-doped ZnO and (Gd, Al) co-doped ZnO films. The contrast of these magnetic grains is around 30 nm to 40 nm, and they cooperate in forming the interaction domain magnetization, which can be described as a ferromagnetic structure [31,32]. All these grains are magnetized by the magnetic tip and generate an attractive force among the grains and tip itself, whose convolution between their magnetic moments produces the characteristic contrast observed in the MFM images. The magnetic force (attractive and repulsive) of the film can be expressed by the following equation [30]:

*F* = −∇E = *µ*_0_ ∫∇ (M_tip_·H_sample_) *dV*_tip_ = *µ*_0_ ∫∇ (M_sample_·H_tip_) *dV*_sample_ (6)

in which M_tip_ is the convolution of the tip magnetic moment, H_sample_ is the sample stray magnetic field, M_sample_ is the convolution of the sample magnetic moment, and H_tip_ is the tip stray magnetic field.

**Figure 7:**
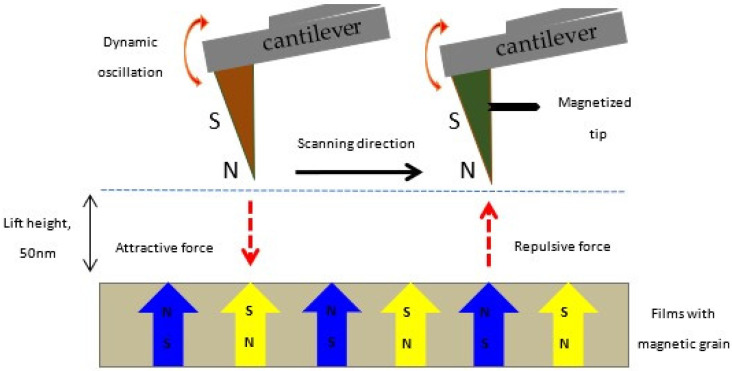
Schematic process for MFM image of ZnO-based DMS films. The appearance of magnetization in films, as well as in the probing tip, is represented by the magnetic poles: North (N)—up and South (S)—down.

The cooperation of the spin polarization between the positive south (S) and negative north (N) of the tip and films is equivalent to the attractive forces, whereas the spin polarization between (N) and (N) are respective to the repulsive forces, as shown in Figure 7. Note that these magnetic poles are used to represent the orientation of the tip magnetization. From Figure 6a, there is no bright and dark contrast in MFM images, indicating that undoped ZnO film is non-magnetic. As opposed to Gd-doped ZnO and (Gd, Al) co-doped ZnO films, a magnetic response was observed in the MFM images as a bright and dark contrast, which suggests the existence of ferromagnetism in doped films. This bright and dark contrast in the images describes the domain magnetic structure pattern by the spin polarization of the (S) and (N) magnetic poles, respectively [33]. The presence of the bright and dark contrast expressed from the magnetic forces indicates the intrinsic ferromagnetism and spin polarization in the films.

**Figure 8:**
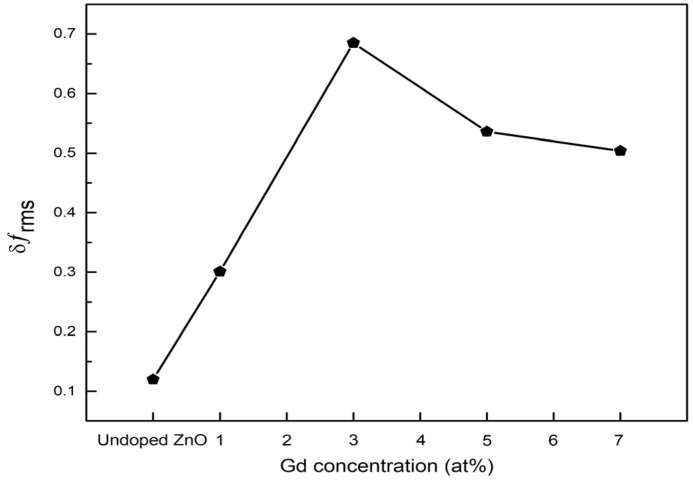
Root mean square values, *δf*_rms_, of MFM images of undoped ZnO and Gd-doped ZnO films.

The presence of the magnetic contrast observed from the MFM images corresponds to the root mean square value, *δf*_rms_, of the MFM images. The *δf*_rms_ of the MFM images can be detailed by the following equation [34]:

(7) $$\delta f_{rms}=\sqrt{\sum \frac{{\left(f_{i}-f_{0}\right)}^{2}}{N-1}}$$

where *f*_i_ is the pixel of the MFM signal, *f*_0_ is the average MFM signal, and N is the total pixel number of the MFM image. Figure 8 shows the *δf*_rms_ of the MFM images corresponding to the undoped ZnO film and Gd-doped ZnO films with various Gd concentrations. Based on the plotted result in Figure 7, the *δf*_rms_ of the MFM images increases until the 3 at% Gd concentration and continuously decreases when the Gd concentration increases up to 7 at%. This implies that the magnetic moment produced by the magnetic contrast intensifies with the addition of Gd into the ZnO lattice [28]. Moreover, with the incorporation of Al into Gd-doped ZnO, the *δf*_rms_ values increase to 9.10 as tabulated in Table 3. As reported by Jin et al., the magnetic contrast is predominantly produced by the magnetic moment, where the higher magnetic contrast of the MFM images results in higher *δf*_rms_ values [35]. Thus, the higher *δf*_rms_ value at 3 at% (Gd, Al) co-doped ZnO indicates that the magnetic moment increases when Al is co-doped into the ZnO. This proves that the incorporation of Al into Gd-doped ZnO improves the intrinsic ferromagnetism and spin polarization of the films.

**Figure 9:**
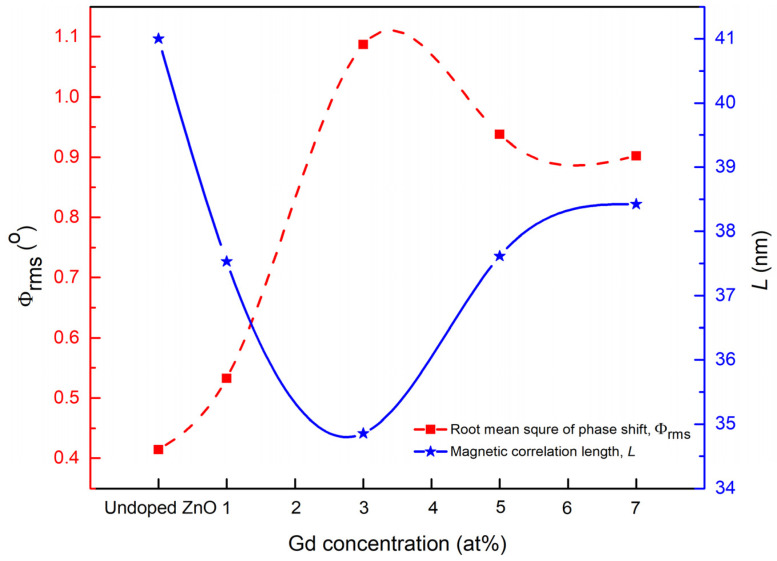
Correlation of root mean square of phase shift, Φ_rms_ and magnetic correlation length, *L*, upon various Gd concentrations.

Based on the observation through MFM images in Figure 6, the domain size resulting in the nanometer range and the variation was almost the same with varied Gd concentrations. The distribution of the domains seems equally proportioned for all the samples with a smaller-sized domain observed at 3 at% Gd and a bigger domain size at 7 at% Gd, while the size of the domain becomes smaller when Al is incorporated into Gd-doped ZnO. In order to know the exact average of the domain size, the magnetic correlation length, *L*, was calculated for the complex uneven interaction of the domain size [32,36]. The XEI software for MFM grain size analysis was used to calculate *L* based on the produced MFM images as the mean values of the lateral size of the domain area. The resulting *L* values indicate the mean of the magnetic domains that are measured on the surface of the sample in the area 1 μm × 1 μm. The calculated mean values of the magnetic correlation *L* for all samples are shown in Table 3. From Figure 9, the lowest *L* value was at 3 at%, which was 34.87 nm, as the values show a decrement with increasing Gd concentration. However, by increasing the Gd concentration to 5 at% and 7 at%, the value of *L* started to increase. By the incorporation of Al into Gd-doped ZnO, the magnetic correlation *L* decreases to 26.42 nm. The results produced can be interpreted by inter-grain exchange coupling occurring among magnetic grains. Commonly, the magnetic correlation length, *L*, is correlated with the inter-grain exchange coupling. Therefore, the lowest *L* values resulting at 3 at% Gd and 3 at% (Gd, Al) were due to the weak transfer of inter-grain coupling between strong magnetic domains, which consequently form smaller magnetic domains. This elucidates that a certain dopant concentration, as well as dopant element, develops the utmost energy of the sample, which is ascribed to the refinement of the magnetic microstructure arising from the weaker inter-grain exchange coupling action.

Furthermore, the phase shift ΔΦ in the midst of the drive voltage and the cantilever response can be determined from the MFM images. The magnetic force gradient distribution is measured using a magnetic coating tip that is placed on top of the surface of the sample by the oscillating motion of the cantilever normal to the sample surface at its resonance frequency. Magnetic attractive and repulsive forces produce a change in the amplitude A and cause a shift of both the phase Φ and the resonance frequency *f*_0_ in such a force. The amplitude A, the phase Φ, and the resonance frequency *f*_0_ are defined as [37,38]:

(8) $$\Delta A=\frac{A_{0\;}Q_{c}}{2\;k_{c}^{2}}{\left(\frac{\partial F_{Z}}{\partial z}\right)}^{2}$$

(9) $$\Delta \Phi \;=\frac{Q_{c}}{\;k_{c}}\frac{\partial F_{Z}}{\partial F_{Z}}$$

(10) $$\Delta f_{0}=-\frac{f_{0}}{\;2k_{c}}\frac{\partial F_{Z}}{\partial F_{Z}}$$

where *k*_c_, *Q*_c_, $A_{0}$, and *f*_0_ are the cantilever spring constant, the cantilever quality factor in air, the amplitude at resonance frequency in air, and the resonance frequency, respectively.

For this study, the drive frequency shifts lead to phase shifts, ΔΦ, which provides an image of the magnetic force gradients. The root mean square value of the phase shift Φ_rms_ of the MFM images indicates the standard deviation of the phase shift ΔΦ within the given scan area and it can be calculated using an equation as follows [39]:

(11) $$\Phi_{\mathrm{rms}}=\surd \frac{\sum_{i=1}^{N}(\;\Phi_{i}-\Phi_{ave})\;2\;}{N}$$

where Φ_ave_ is the average Φ value in the scan area of the MFM image, Φ_i_ is the current ith Φ value, and *N* is the number of points in a given area. 

The calculated Φ_rms_ values of the MFM images of undoped ZnO and Gd-doped ZnO with Gd = 1, 3, 5, 7 at% are as listed in Table 3. The largest Φ_rms_ value was found at 3 at%, in which the result is in good concurrence with the magnetic correlation length *L* and inversely proportional to each other. This is associated with the improvement of the ultimate energy product through enhanced magnetic domain refinement. Thus, it is evident that 3 at% has better magnetic properties as it has a smaller domain size with a shorter magnetic correlation length *L* and a larger phase shift Φ_rms_ among other samples. Moreover, the magnetic properties were improved when co-doped with Al, where the Φ_rms_ is 1.678, which is larger when compared to 3 at% Gd. These observations are similar to the lowest magnetic correlation length, *L*, and highest *δf*_rms_ value obtained when co-doped with Al, which results in the enhancement of magnetic properties. From this result, the refinement and uniformity of the small magnetic domain size, magnetic microstructure, and weak inter-grain exchange coupling play vital roles in the good performance of magnetic properties [31,39].

**Figure 10:**
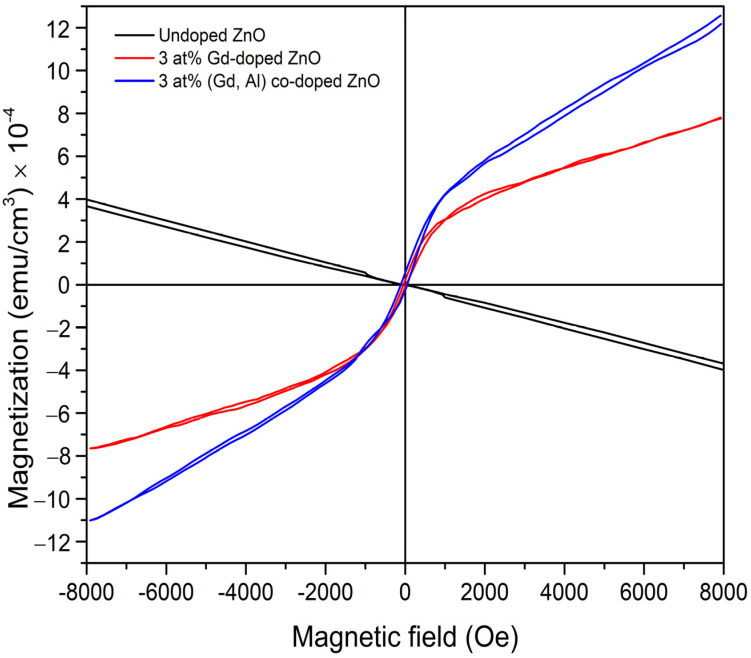
M-H curves of undoped ZnO, 3 at% Gd-doped ZnO, and 3 at% (Gd, Al) co-doped ZnO.

Measuring the magnetization as a function of the magnetic field, (M-H) curves were conducted using VSM at room temperature in the magnetic field range from −8 kOe to +8 kOe. The magnetization data presented in M-H curves were corrected by subtracting the diamagnetic background of the substrate. The room-temperature M-H curves of the undoped ZnO, 3 at% Gd-doped ZnO, and 3 at% (Gd, Al) co-doped ZnO are shown in Figure 10. The M-H curve for undoped ZnO film shows a diamagnetic behavior which is in line with the MFM image, indicating that the film is non-magnetic, whereas the samples with 3 at% Gd-doped ZnO and 3 at% (Gd, Al) co-doped ZnO exhibit magnetic behavior with clear hysteresis and saturation magnetization (M_s_) at room temperature. The M-H curves demonstrate the increase in saturation magnetization in a sample of 3 at% (Gd, Al) co-doped ZnO compared to a sample with only Gd-doped ZnO. The M_s_ values were 7.78 × 10^−4^ emu/cm^3^ and 12.56 × 10^−4^ emu/cm^3^ for Gd-doped ZnO and (Gd, Al) co-doped ZnO, respectively. The incorporation of extra shallow donors by purposely co-doping GdZnO with shallow donor Al resulted in an increase in M_s_, indicating the improvement in carrier concentration, as measured by the Hall effect, which is further discussed in the next section. The mobile carriers produced carrier-mediated exchange interactions between conducting electrons and local spin-polarized electrons linked to the Gd and Al ions, thus resulting in the extended ferromagnetism of the film [40]. A similar study reported by Poornaprakash et al. [40] shows the enhanced ferromagnetism by induced Al as co-doping, while H. Sun et al. [41] found carrier-mediated ferromagnetism in (Al, Co) co-doped ZnO, where the co-doping caused an increase in carrier concentration. This result proves that co-doping is a promising way to enhance the ferromagnetic properties of ZnO-based DMS systems.

### 3.5. Electrical Properties

**Figure 11:**
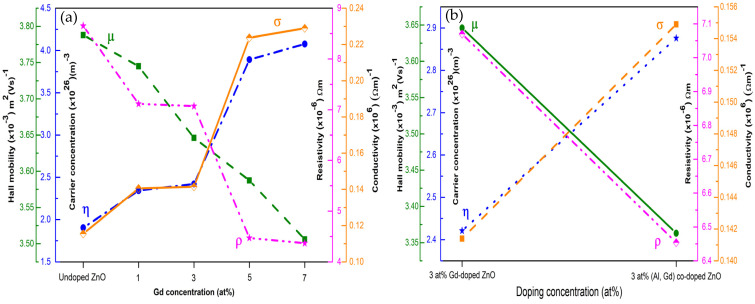
The variation of hall mobility, carrier concentration, resistivity, and conductivity as a function of (**a**) undoped ZnO and Gd-doped ZnO films, (**b**) 3 at% Gd-doped ZnO and 3 at% (Gd, Al) co-doped ZnO films.

The variation in carrier concentration, hall mobility, resistivity, and conductivity as a function of different Gd concentrations is shown in Figure 11a, while Figure 11b shows the comparison of electrical properties between 3 at% Gd-doped ZnO and 3 at% (Gd, Al) co-doped ZnO films. All the obtained values are listed in Table 4. It was found that the carrier type is an n-type conductivity generated for all the films. The plotted graph in Figure 11 shows the electrical resistivity decreased along with increasing Gd concentrations. The decrease in resistivity with Gd concentration could possibly be because of the capture of free electrons in the ZnO lattice via the empty lower energy 3d Zn states. These results also correspond well with the XRD data, where the decrease in crystallite size caused by the smaller scattering of grain boundary resulted in low electrical resistivity [17]. Anand et al. also obtained similar electrical resistivity values of roughly 10^−4^ Ωcm [17]. The electrical conductivity was observed to be decreasing with the increment in Gd concentration. The same observations were achieved for 3 at% (Gd, Al) co-doped ZnO compared to 3 at% Gd. These results prove that the electrical conductivity in ZnO can be improved by incorporating doping elements, i.e., Gd and Al.

The carrier concentration improved with the addition of Gd concentration and Al co-doping. This is due to the substitution of Gd^3+^ and Al^3+^ ions into the Zn^2+^ sites generating more free electrons in the films. The interstitial place of Gd^3+^ and Al^3+^ ions within the ZnO matrix also enhanced the carrier concentration. An opposite trend with respect to the carrier concentration was discovered for carrier mobility. This observed trend was due to the ionized impurity scattering mechanism. The carrier mobility decreases at higher levels of Gd doping concentration and, with the addition of Al, was also affected by the low crystalline structure discovered through those films. These results can be confirmed through XRD data, as the decrement in the peak intensity is observed when ZnO films were co-doping with Gd and Al. Through a comparison of the previous literature with the current work tabulated in Table 5, it is concluded that co-doping Al into rare-earth doped DMSs results in the enhancement of the carrier concentration, as well as other electrical properties.

## 4. Conclusions

Undoped ZnO, (1, 3, 5, and 7 at%) Gd-doped ZnO, and 3 at% (Gd, Al) co-doped ZnO films were successfully synthesized by a co-reactive sputtering method. The structural analyses showed that Gd and Al ions are successfully incorporated into the host ZnO lattice at the Zn^2+^ site. No secondary phase was observed in the Gd and (Gd, Al) co-doped ZnO films. The presence of Gd and Al in the ZnO matrix was confirmed by EDS analysis for Gd-doped ZnO and (Gd, Al) co-doped ZnO films. The UV–Vis results show that the optical transmittance is above 80% for all films and the optical band gap shows a redshift with increasing Gd concentration. The optical band gap of 3 at% (Gd, Al) was also found to be redshifted from the sample with 3 at% Gd. The MFM measurement showed the existence of intrinsic ferromagnetism and spin polarization in Gd and (Gd, Al) co-doped ZnO films based on the appearance of a bright–dark contrast in the MFM images. The results obtained from the MFM analysis exhibit the enhancement of magnetic properties for 3 at% Gd and 3 at% (Gd, Al) co-doped ZnO films due to the smaller domain size with a shorter magnetic correlation length L, a larger phase shift Φ_rms_, and the highest value of *δf*_rms_. Room-temperature M-H curves further confirm the enhancement of the saturation magnetization of a Gd-doped ZnO sample that incorporated a shallow donor Al. The carrier concentration and electrical conductivity improves with increasing Gd concentration, while the hall mobility and resistivity decrease as low as 3.5 × 10^−3^ m^2^/V·s and 4.3 × 10^−6^ Ω·m, respectively. The similar trend of results obtained for 3 at% (Gd, Al) co-doped ZnO compared to 3 at% Gd-doped ZnO, indicates that Al incorporation into ZnO improves electrical properties. The findings reveal that 3 at% Gd with the addition of 3 at% Al improves the physical, magnetic, and electrical properties and might lead to the enhancement of the ferromagnetism at room temperature, which may open new pathways in spintronic applications.

## Figures and Tables

**Table 1:** Structural properties of undoped ZnO, Gd-doped ZnO films, and 3 at% (Gd, Al) co-doped ZnO films.

Sample	Orientation Peak	Crystallite Size, D (nm)	FWHM (2 Th)	Intensity (cts)	Dislocation Density δ (10^−3^ nm^−2^)	Microstrain ε (10^−3^ lin^−2^ m^−2^)
Undoped ZnO	(002)	15.65	0.5312	15,823.7	4.08	7.53
1 at% Gd	(002)	6.25	1.3299	6386.4	23.48	18.95
3 at% Gd	(002)	10.82	0.7673	7931.6	7.82	10.97
5 at% Gd	(002)	3.65	2.2761	4418.1	68.85	32.58
7 at% Gd	(002)	2.71	3.0619	3532.2	124.75	44.13
3 at% (Gd, Al)	(002)	11.08	0.7502	7274.5	7.47	10.66

**Table 2:** Elemental composition of undoped ZnO, various Gd concentrations of Gd-doped ZnO, and (Gd, Al) co-doped ZnO films.

Sample	Al (at%)	Gd (at%)	Zn (at%)	O (at%)
Undoped ZnO	NA	NA	38.92	61.08
1 at% Gd	NA	1.14	43.08	55.78
3 at% Gd	NA	3.09	37.58	59.33
5 at% Gd	NA	5.11	38.62	56.27
7 at% Gd	NA	7.27	35.24	60.19
3 at% (Gd, Al) co-doped ZnO	3.00	3.00	41.80	52.14

**Table 3:** The magnetic properties of undoped ZnO, (1, 3, 5, 7 at%) Gd-doped ZnO, and 3 at% (Gd, Al) co-doped ZnO films.

Sample	Φ_rms_ (^o^)	*L* (nm)	∂*f*_rms_
Undoped ZnO	0.41	41.00	0.12
1 at% Gd	0.53	37.53	0.30
3 at% Gd	1.09	34.87	0.69
5 at% Gd	0.94	37.62	0.54
7 at % Gd	0.90	38.43	0.50
(Gd, Al) co-doped ZnO	1.68	26.42	9.10

**Table 4:** The electrical parameters of undoped ZnO, Gd-doped ZnO, and 3 at% (Gd, Al) co-doped ZnO films.

Sample	Carrier Concentration ×10^26^ (1/m^3^)	Hall Mobility ×10^−3^ (m^2^/Vs)	Resistivity ×10^−6^ Ωm	Conductivity ×10^6^ (Ωm)^−1^
Undoped ZnO	1.904	3.788	8.653	0.1155
1 at% Gd	2.343	3.745	7.115	0.1405
3 at% Gd	2.421	3.646	7.070	0.1414
5 at% Gd	3.892	3.587	4.471	0.2237
7 at % Gd	4.077	3.506	4.366	0.2290
(Gd, Al) co-doped ZnO	2.876	3.363	6.454	0.1549

**Table 5:** Literature of carrier concentration of ZnO-based DMS films.

Sample	Carrier Concentration (1/m^3^)	Reference
1.5 at% Gd	2.12 × 10^17^	[42]
3 at% (Al, Cu) co-doped ZnO	3.87 × 10^15^	[43]
1.5 at% (Eu, Al) co-doped ZnO	4.42 × 10^19^	[3]
5.2 at% (Al, Co) co-doped ZnO	1.95 × 10^18^	[41]
1.5 at% (Nd, Al) co-doped ZnO	3.96 × 10^19^	[2]
3 at% (Gd, Al) co-doped ZnO	2.88 × 10^26^	This work

## Data Availability

Data are contained within the article.
